# Salicylic acid improves *Nasturtium officinale* phytoremediation capability for cadmium-contaminated paddy soils

**DOI:** 10.3389/fpls.2022.1059175

**Published:** 2022-11-24

**Authors:** Yangxia Zheng, Ran Zhang, Ying Zhu, Qiaoman Ao, Han Liu, Aihui Li, Lijin Lin, Li Wang

**Affiliations:** ^1^ College of Horticulture, Sichuan Agricultural University, Chengdu, Sichuan, China; ^2^ Institute of Pomology and Olericulture, Sichuan Agricultural University, Chengdu, Sichuan, China; ^3^ College of Animal Science and Technology, Sichuan Agricultural University, Chengdu, Sichuan, China

**Keywords:** salicylic acid, *Nasturtium officinale*, cadmium, growth, phytoremediation

## Abstract

**Introduction:**

Cadmium (Cd) contamination is a severe problem in paddy soils that has affected crops’ safety. The present study aimed at remediating Cd-contaminated paddy soil by improving the phytoremediation capability of aquatic accumulator plants.

**Methods:**

We conducted an experiment to investigate the effects of salicylic acid (SA) on the growth and Cd phytoremediation capability of the aquatic accumulator plant *Nasturtium officinale*.

**Results:**

SA with the concentrations of 100, 150, and 200 mg/L increased the root and shoot biomass of *N. officinale*, while only 150 mg/L increased the chlorophyll a and b contents. SA increased the activities of peroxidase and catalase of *N. officinale* to a great extent, but decreased the superoxide dismutase activity and soluble protein content. SA also increased the root Cd content, shoot Cd content, root Cd extraction, and shoot Cd extraction to a large extent. At concentrations of 100, 150, and 200 mg/L, SA increased the shoot Cd extraction by 17.59%, 47.16%, and 43.27%, respectively, compared with the control. Moreover, SA concentration had a quadratic polynomial regression relationship with the root Cd extraction and shoot Cd extraction. The correlation and grey relational analyses revealed that root Cd extraction, shoot biomass, and root biomass were closely associated with shoot Cd extraction of *N. officinale*.

**Conclusion:**

Thus, our results suggest that SA promoted the growth and improved the phytoremediation (extraction) capability of *N. officinale*, and 150 mg/L SA was the most suitable concentration.

## Introduction

The major pollutants in the soil are heavy metals. The major heavy metals causing soil contamination is cadmium (Cd), which far exceeds the contamination due to other heavy metal elements (Riaz et al., 2021). As a non-essential element, excessive Cd causes toxicity and affects the growth of plants (Garg et al., 2015; Vatehová et al., 2015). Cadmium has strong biotoxicity and transferability in soil and can be absorbed by plants (Anna et al., 2015). After entering the human body through the food chain, Cd causes toxic effects on some internal organs and damages the human body’s immune system and reproductive system (Xie and Xu, 2003). In recent years, paddy fields have been severely contaminated by Cd, threatening human health (Song et al., 2015; Zhang et al., 2020). So, it’s important to remediate Cd-contaminated soil, specifically the Cd-contaminated paddy soils. Many methods have been used to remediate Cd-contaminated soils (Mahmoud et al., 2021; Wang et al., 2021). Among these, phytoremediation is one of the best biological methods used for extracting, fixing, and concentrating the Cd from the soil through Cd-hyperaccumulators; it is an environmentally friendly approach (Guo and Yang, 2015; Shaheen and Rinklebe, 2015). However, the screened Cd-hyperaccumulators have the problems of low biomass and slow growth, which limit the phytoremediation effects (Guo and Yang, 2015). Therefore, it is necessary to improve the phytoremediation capabilities of these known hyperaccumulators.

Application of plant hormones can be used as a new method to improve the phytoremediation capabilities of hyperaccumulators (Rostami and Azhdarpoor, 2019). The plant hormone salicylic acid (SA) is an intracellular signaling molecule in plants (Kawano and Bouteau, 2013). SA mediates the plants to adapt the abiotic stress (Azooz and Youssef, 2010) and improves plant resistance by increasing photosynthesis and antioxidant enzyme activity, thereby slowing down the damage caused by adversity (Song et al., 2014). Under heavy metal stress, SA improves the resistance of plants (Song et al., 2014; Sofy et al., 2020). SA treatment increased the antioxidant enzyme activity of cauliflower under Cd stress and alleviated the cell damage under Cd stress (Chen, 2009). The peroxidation of barley root tips was inhibited when treated with SA under Cd stress (Tamás et al., 2015). For rice, SA treatment increased its antioxidant enzyme activity and decreased the H_2_O_2_ content to enhance the resistance to Cd stress (Guo et al., 2007); this also decreases the rice Cd content (Majumdar et al., 2020; Pan et al., 2021). Various other studies have shown similar effects of SA on mung beans, kidney beans and tomatoes under Cd stress (Tissa et al., 2000; Khan et al., 2021). In addition, SA also has demonstrated the effects on hyperaccumulators. SA increased the Cd content and transportation factor and improved the phytoremediation capability of *Helianthus tuberosus* to Cd (Chen et al., 2011). So, SA could improve the tolerance of plants to Cd stress and enhance the phytoremediation capability of hyperaccumulators; however, there are few studies on the response of the SA-treated hyperaccumulators.

*Nasturtium officinale* is a genus of cruciferous watercress plants emerging as a Cd-accumulator (Lin et al., 2015). *Nasturtium officinale* has strong vitality, vigorous growth, wide distribution, and short growth cycle (Chen et al., 2006). However, compared with other Cd-hyperaccumulators (Wei et al., 2004; Zhang et al., 2011b; Zhang et al., 2013), the *N. officinale* phytoremediation capability needs to be improved. Therefore, the present study aimed to improve the phytoremediation capability of *N. officinale* and analyzed the effects of SA on the growth and Cd accumulation of *N. officinale*. The aim of this study was to screen the optimal SA concentration that could improve the *N. officinale* phytoremediation capability and provide a reference for remedying the Cd-contaminated paddy fields.

## Materials and methods

### Materials

The *N. officinale* plants were collected from the farmland of Ya’an Campus of Sichuan Agricultural University (29° 59′ N, 102° 59′ E) as reported by the previous study of Tang et al. (2022), and 10 cm long shoot tips were cut for cuttings. Soil samples with no detectable Cd were collected, and their physicochemical properties were the same as Tang et al., 2022. The analytically pure SA used in this study was purchased from the Chengdu Kelong Chemical Co., Ltd., Chengdu, China.

### Experimental design

The experiment was carried out in a greenhouse at the Chengdu Campus of Sichuan Agricultural University (30°42′N, 103°51′E) from August to October 2020. The soil samples (3.0 kg) were put into a plastic pot and treated with the pure analytical CdCl_2_·2.5H_2_O to obtain a final soil Cd concentration of 5 mg/kg (Tang et al., 2022) in August 2020. Then, the soil was watered every day for a month to make it flooded. In September 2020, three uniform cuttings of *N. officinale* were planted in each pot, with the lower 5 cm inside the soil and the upper 5 cm above the soil. These pots were watered, maintaining a water depth of 2 cm above the soil surface. One week later, the SA solution (0, 100, 150, 200, and 250 mg/L) was sprayed on both sides of the leaves of *N. officinale* until the SA solution started dripping (Liu et al., 2022). The SA solution was resprayed after a week. Triplicates (three pots) were maintained per treatment. The pots were watered daily to maintain a water depth of 5 cm above the soil surface from the first time SA treated until harvest.

### Determination of plant parameters

Thirty days after the first SA spray (October 2020), the fourth and fifth mature leaves of *N. officinale* were collected to determine the photosynthetic pigment (chlorophyll *a*, chlorophyll *b*, and carotenoid) contents, antioxidant enzyme [peroxidase (POD), superoxide dismutase (SOD), and catalase (CAT)] activities, and soluble protein content as reported by Hao et al. (2004); Lin et al. (2020)and Tang et al. (2022) and . After that, the plants were uprooted, cleaned, and dried as described by Lin et al. (2020). The root and shoot (dry weight) were weighed using an electronic balance. The dried samples were finely ground and digested (Lin et al., 2020), and the root and shoot Cd contents were determined using an ICAP6300 ICP spectrometer (Thermo Scientific, Waltham, MA, USA). The root Cd extraction and the shoot Cd extraction were calculated as follows: root Cd extraction = root Cd content × root biomass, and shoot Cd extraction = shoot Cd content × shoot biomass (Zhang et al., 2010). Finally, the pot soil was collected, air-dried, and sieved to determine the soil pH and exchangeable Cd concentration using a pH meter and an ICAP6300 ICP spectrometer, respectively (Bao, 2000; Tang et al., 2022).

### Statistical analysis

The data were analyzed using SPSS 20.0.0 software (IBM, Chicago, IL, USA) with three replicates per treatment. Data were normalized and subjected to a homogeneity test, followed by a one-way analysis of variance (ANOVA) and Duncan’s Multiple Range Test (*P* < 0.05). The quadratic polynomial relationship between SA concentration and the Cd extraction was analyzed using regression analysis. The Pearson’s correlation was used to analyze the relationships among the different indicators. The grey relational analysis was used to assess the relationships of the different indicators with the shoot Cd extraction (Wang, 2019; Ma et al., 2022).

## Results

### *N. officinale* biomass

**Figure 1:**
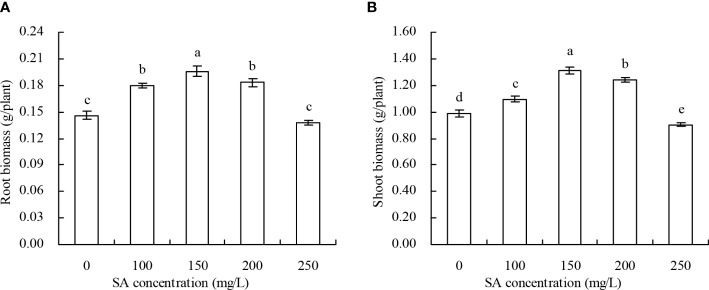
Biomass of *Nasturtium officinale*. **(A)** Root biomass; **(B)** shoot biomass. Data shown are mean values ( ± SE) of three replicates. Different lowercase letters indicate significant differences among the treatments (Duncan’s Multiple Range Test, *P* < 0.05).

The biomass of *N. officinale* increased with the increase in SA concentration to 150 mg/L and decreased with the SA concentration above 150 mg/L (**Figures 1A, B**). Compared with the control, 100, 150, and 200 mg/L SA increased by 23.29%, 34.25%, and 25.34% of the root biomass, respectively, and by 11.05%, 32.96%, and 25.66% of the shoot biomass, respectively; however, there were no significant impacts of root and shoot biomass treated with 250 mg/L SA.

### *N. officinale* photosynthetic pigment content

Compared with the control, only 150 mg/L SA increased the chlorophyll *a* and chlorophyll *b* contents in *N. officinale* by 13.30% and 17.29%, respectively (**Table 1**). However, SA at other concentrations had no significant effect. SA at 100 and 200 mg/L decreased the chlorophyll *a*/*b* by 0.128 and 0.150, respectively, compared with the control, but at 150 and 250 mg/L had no significant impact. Meanwhile, for the carotenoid content, SA had no significant impact.

**Table 1:** Photosynthetic pigment content in *Nasturtium officinale*.

SA concentration(mg/L)	Chlorophyll *a*(mg/g)	Chlorophyll *b*(mg/g)	Chlorophyll *a*/*b*	Carotenoid(mg/g)
0	0.361 ± 0.010bc	0.133 ± 0.003bc	2.721 ± 0.006ab	0.059 ± 0.003ab
100	0.371 ± 0.007bc	0.143 ± 0.006ab	2.593 ± 0.049c	0.064 ± 0.001a
150	0.409 ± 0.004a	0.156 ± 0.001a	2.630 ± 0.002bc	0.065 ± 0.001a
200	0.376 ± 0.008b	0.146 ± 0.005ab	2.571 ± 0.040c	0.063 ± 0.002a
250	0.349 ± 0.006c	0.123 ± 0.001c	2.824 ± 0.022a	0.055 ± 0.002b

Data shown are mean values ( ± SE) of three replicates. Different lowercase letters indicate significant differences among the treatments (Duncan’s Multiple Range Test, P < 0.05).

### *N. officinale* antioxidant enzyme activity and soluble protein content

With the increase in SA concentration, the POD and CAT activities of *N. officinale* increased, while the SOD activity and soluble protein content decreased (**Table 2**). Compared with the control, SA at 250 mg/L increased the POD activity by 11.40%, and at the concentrations of 150, 200, and 250 mg/L increased the CAT activity by 26.21%, 39.37%, and 54.10%, respectively. SA at 150, 200, and 250 mg/L concentrations decreased the SOD activity by 10.00%, 14.34%, and 17.14%, respectively, and the concentrations of 100, 150, 200, and 250 mg/L SA decreased the soluble protein content by 12.95%, 31.65%, 41.34%, 43.56%, respectively, compared with their respective control.

**Table 2:** Antioxidant enzyme activity and soluble protein content of *Nasturtium officinale*.

SA concentration(mg/L)	POD activity(U/g/min)	SOD activity(U/g)	CAT activity(mg/g/min)	Soluble protein content(mg/g)
0	1123 ± 12.50b	228.1 ± 0.75a	2.205 ± 0.066d	66.42 ± 2.23a
100	1138 ± 12.01b	213.6 ± 1.18ab	2.253 ± 0.055d	57.82 ± 2.12b
150	1165 ± 13.00b	205.3 ± 5.41bc	2.783 ± 0.103c	45.40 ± 2.19c
200	1178 ± 16.03b	195.4 ± 5.52c	3.073 ± 0.043b	38.96 ± 1.91cd
250	1251 ± 27.08a	189.0 ± 6.37c	3.398 ± 0.094a	37.49 ± 0.98d

Data shown are mean values ( ± SE) of three replicates. Different lowercase letters indicate significant differences among the treatments (Duncan’s Multiple Range Test, P < 0.05).

### *N. officinale* Cd content

**Figure 2:**
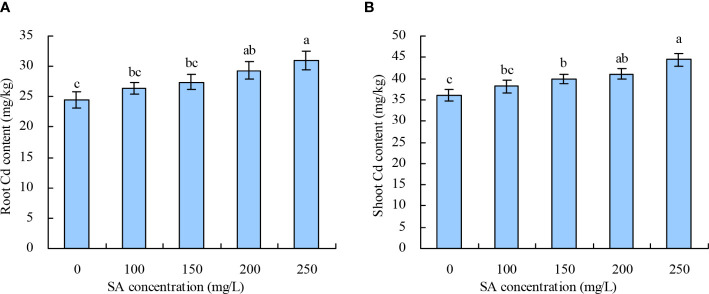
Cd content in *Nasturtium officinale*. **(A)** Root Cd content; **(B)** shoot Cd content. Data shown are mean values ( ± SE) of three replicates. Different lowercase letters indicate significant differences among the treatments (Duncan’s Multiple Range Test, *P* < 0.05).

With the increase in SA concentration, the root and shoot Cd contents in *N. officinale* increased (**Figures 2A, B**). Compared with the control, 200 and 250 mg/L SA increased the root Cd content by 19.99% and 26.45%, respectively, while other concentrations of SA had no significant impact. SA at 150, 200, and 250 mg/L increased the shoot Cd content by 10.69%, 14.02%, and 23.49%, respectively, while 100 mg/L had no significant impact compared with the control.

### *N. officinale* Cd extraction

**Figure 3:**
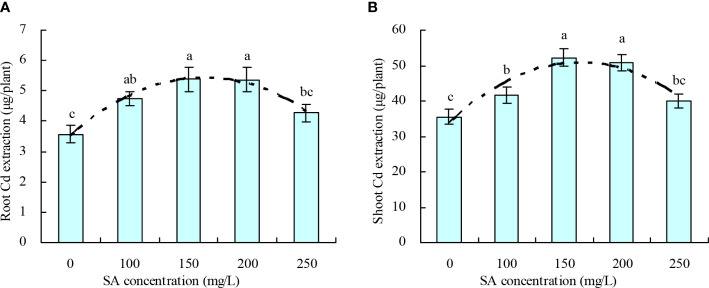
Cd extraction by *Nasturtium officinale*. **(A)** Root Cd extraction; **(B)** shoot Cd extraction. Data shown are mean values ( ± SE) of three replicates. Different lowercase letters indicate significant differences among the treatments (Duncan’s Multiple Range Test, *P* < 0.05). Root Cd extraction, Cd content in roots × root biomass; shoot Cd extraction, Cd content in shoots × shoot biomass.

The root and shoot Cd extractions of *N. officinale* increased with the increase in SA concentration to 150 mg/L and then decreased above 150 mg/L (**Figures 3A, B**). Compared with the control, 100, 150, and 200 mg/L SA increased the root Cd extraction by 33.04%, 50.53%, 50.36%, respectively, and increased the shoot Cd extraction by 17.59%, 47.16%, and 43.27%, respectively; however, 250 mg/L SA had no significant impact on the root and shoot Cd extractions. Moreover, the SA concentration displayed a quadratic polynomial regression relationship with both the root Cd extraction (y = -7.981E-5x^2^ + 0.024x + 3.489, R^2^ = 0.830, *P* = 0.000) and shoot Cd extraction (y = -0.001x^2^ + 0.197x + 34.178, R^2^ = 0.676, *P* = 0.001).

### Soil pH and exchangeable Cd concentration

**Figure 4:**
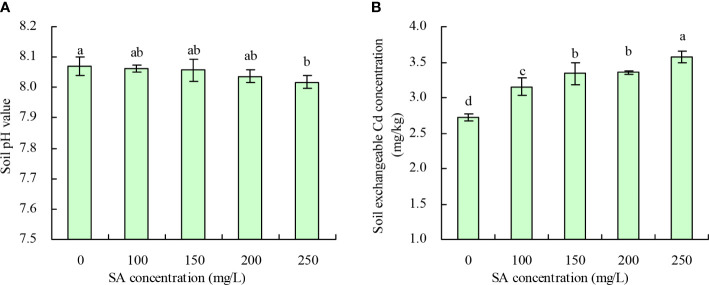
Soil pH value and soil exchangeable Cd concentration. **(A)** Soil pH value; **(B)** Soil exchangeable Cd concentration. Data shown are mean values ( ± SE) of three replicates. Different lowercase letters indicate significant differences among the treatments (Duncan’s Multiple Range Test, *P* < 0.05).

Compared with the control, SA at 250 mg/L decreased the soil pH by 0.053, while 100, 150, and 200 mg/L concentrations had no significant effect (**Figure 4A**). Meanwhile, the soil exchangeable Cd concentration increased with an increase in SA concentration (**Figure 4B**). Compared with the control, the concentrations of 100, 150, 200, and 250 mg/L SA increased the soil exchangeable Cd concentration by 15.92%, 22.66%, 23.27%, and 31.23%, respectively.

### Correlation and grey relational analyses

The Pearson’s correlation analysis showed that both the root biomass and shoot biomass were positively correlated (*P* < 0.01) with the chlorophyll *a* content, chlorophyll *b* content, and carotenoid content, and negatively correlated (*P* < 0.01) with the chlorophyll *a*/*b* ratio (**Table 3**). Both the root Cd content and shoot Cd content were positively correlated (*P* < 0.01) with the POD activity, CAT activity, and soil exchangeable Cd concentration, and negatively correlated with the SOD activity and soluble protein content (*P* < 0.01), and soil pH (0.01 ≤ *P* < 0.05). Both the root Cd extraction and shoot Cd extraction were positively correlated with the root biomass, shoot biomass, chlorophyll *a* content, chlorophyll *b* content, and carotenoid content (0.01 ≤ *P* < 0.05), and negatively correlated with the chlorophyll *a*/*b* and soluble protein content (0.01 ≤ *P* < 0.05). The shoot Cd extraction was positively correlated with the root Cd extraction (*P* < 0.01), while the soil exchangeable Cd concentration was negatively correlated with soil pH (*P* < 0.01).

**Table 3:** Correlations among the different indicators.

Indicators	Root biomass	Shoot biomass	Chlorophyll *a* content	Chlorophyll *b* content	Chlorophyll *a*/*b*	Carotenoid content	POD activity	SOD activity	CAT activity	Soluble protein content	Root Cd content	Shoot Cd content	Soil pH value	Soil exchangeable Cd concentration	Root Cd extraction	Shoot Cd extraction
Root biomass																
Shoot biomass	0.955**															
Chlorophyll *a* content	0.822**	0.864**														
Chlorophyll *b* content	0.911**	0.914**	0.943**													
Chlorophyll *a*/*b*	-0.838**	-0.779**	-0.614*	-0.841**												
Carotenoid content	0.893**	0.830**	0.698**	0.842**	-0.878**											
POD activity	-0.345	-0.297	-0.292	-0.419	0.547*	-0.597*										
SOD activity	-0.053	-0.085	0.065	0.112	-0.183	0.245	-0.864**									
CAT activity	-0.139	-0.029	-0.121	-0.220	0.340	-0.363	0.851**	-0.905**								
Soluble protein content	-0.158	-0.233	-0.059	-0.005	-0.095	0.107	-0.792**	0.955**	-0.941**							
Root Cd content	-0.057	-0.027	-0.244	-0.277	0.268	-0.269	0.826**	-0.927**	0.921**	-0.939**						
Shoot Cd content	-0.143	-0.114	-0.254	-0.337	0.397	-0.354	0.876**	-0.914**	0.934**	-0.919**	0.983**					
Soil pH value	0.254	0.198	0.185	0.228	-0.277	0.375	-0.707**	0.566*	-0.595*	0.555*	-0.520*	-0.556*				
Soil exchangeable Cd concentration	0.138	0.131	0.086	0.024	0.119	-0.127	0.788**	-0.878**	0.832**	-0.889**	0.837**	0.858**	-0.632*			
Root Cd extraction	0.863**	0.848**	0.605*	0.672**	-0.619*	0.658**	0.097	-0.514	0.341	-0.615*	0.451	0.360	-0.021	0.526*		
Shoot Cd extraction	0.840**	0.900**	0.701**	0.714**	-0.566*	0.628*	0.098	-0.482	0.383	-0.624*	0.406	0.329	-0.048	0.491	0.963**	

*N* = 15. **: Correlation is significant at the 0.01 level (two-tailed test). *: Correlation is significant at the 0.05 level (two-tailed test).

**Figure 5:**
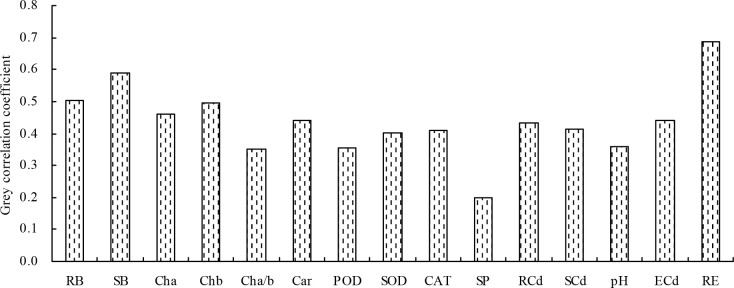
Grey correlation coefficients of the different indicators with the shoot Cd extraction. RB, root biomass; SB, shoot biomass; Cha, chlorophyll *a* content; Chb, chlorophyll *b* content; Cha/b, chlorophyll *a*/*b*; Car, carotenoid content; POD, POD activity; SOD, SOD activity; CAT, CAT activity; SP, soluble protein content; RCd, root Cd content; SCd, shoot Cd content; pH, soil pH value; ECd, soil exchangeable Cd concentration; RE, root Cd extraction.

Finally, the grey relational analysis revealed that the grey correlation coefficients of the different indicators with the shoot Cd extraction ranged from 0.198 to 0.687, indicating that the shoot Cd extraction was correlated with these indicators (**Figure 5**). The root Cd extraction, root biomass, and shoot biomass had higher grey correlation coefficients (>0.500) with the shoot Cd extraction; these indicators were identified as closely associated with the shoot Cd extraction in the following order: root Cd extraction > shoot biomass > root biomass.

## Discussion

The plant hormone SA promotes the growth of plants at an optimal concentration but inhibits plant growth at higher concentrations (Kawano and Bouteau, 2013). Under heavy metal stress, SA can induce the increase in activity of H^+^-ATPase and alleviate the inhibitory effect of this stress on the absorption of mineral elements (Shi and Zhu, 2008). Under Cd stress, SA alleviated the Cd stress and promoted the growth of maize (Krantev et al., 2008). In this study, SA at 100, 150, and 200 mg/L increased the *N. officinale* biomass, while at 250 mg/L decreased the biomass. These results are consistent with previous reports (Kawano and Bouteau, 2013; Liu et al., 2022), which showed that SA at 100 to 200 mg/L promoted the growth of *N. officinale* and at 250 mg/L inhibited. The reason is that SA can not only enhance the root vitality, and promote the absorption of nutrients by roots, but also affect cell division by regulating the content of some hormones in plants (Chavoushi et al., 2020). This promotion effect of SA may be related to the increase in plant resistance to Cd (Pál et al., 2005; Zawoznik et al., 2007; Kováčik et al., 2009).

Typically, Cd stress changes the chloroplast ultrastructure and affects the net photosynthetic rate and leaf transpiration, reducing the photosynthesis of non-hyperaccumulator plants (Souza et al., 2011). Meanwhile, SA alleviates the Cd stress inhibition of photosynthetic parameters by protecting the photosynthetic pigments (Popova et al., 2009; Shi et al., 2009). SA treatment increased the chlorophyll content and enhanced the resistance of potatoes to Cd stress (Li et al., 2019). Similar results were observed in ryegrass too (Bai et al., 2015). In this study, SA at 150 mg/L increased the chlorophyll *a* and *b* contents in *N. officinale* under Cd-contaminated conditions but at 100 and 200 mg/L decreased the chlorophyll *a*/*b*. SA at other concentrations had no significant impact on the contents of chlorophyll *a*, *b*, or chlorophyll *a*/*b*. Meanwhile, none of the SA concentrations significantly influenced the carotenoid content. SA helps the plants to uptake and transport nutrients and thus increases the photosynthetic pigment content (Krantev et al., 2008). These results differ from the effects of SA on *Cyphomandra betacea* (Liu et al., 2022), indicating that different plants respond differently to SA concentrations.

In plants, H_2_O_2_ acts as a second messenger for growth, mediating various physiological and biochemical processes; however, excessive H_2_O_2_ induces oxidative stress (Zhang, 2018). SA and H_2_O_2_ play significant roles in plant stress resistance and have a specific interaction with each other (Zhang et al., 2011a; Zhang, 2018). Under Cd stress, SA removes the reactive oxygen species (ROS) by regulating the antioxidant enzyme activities (Zhang et al., 2011a). In this study, 250 mg/L SA increased the POD activity of *N. officinale* under Cd-contaminated condition, and 150, 200, and 250 mg/L SA increased the CAT activity. These results are not completely consistent with the previous report on *Cyphomandra betacea* (Liu et al., 2022) but consistent with the observations in pea (Popova et al., 2009; Khan et al., 2021), indicating that SA has different impacts on the various plants’ antioxidant system. On the other hand, SA inhibits the activity of key enzymes, including POD, polyphenol oxidase, and phenylalanine ammonia lyase, to slow down lignin synthesis, delaying senescence (Wu et al., 2006). These results reflected that SA improved the resistance of *N. officinale* to Cd. Besides, SA could alleviate oxidative damage under Cd-contaminated conditions. In addition, SA at 150, 200, and 250 mg/L concentrations decreased the SOD activity of *N. officinale*, and at 100, 150, 200, and 250 mg/L concentrations decreased the soluble protein content, which is not consistent with the previous reports (Wang et al., 2009; Liu et al., 2022). Because plants synthesize Cd-containing free amino acids, decreasing soluble protein content; however, a few important soluble proteins may not be seriously affected (Krantev et al., 2008), which needs further study.

Under high pH (pH > 6.0), the soil Cd exists as insoluble compounds formed through complexation, chelation, and precipitation, thereby reducing the soil available Cd concentration. With the decrease in pH, other forms of soil Cd are easily converted into exchangeable Cd, increasing the soil available Cd concentration (Dou et al., 2020). In this experiment, only 250 mg/L SA decreased the soil pH, indicating that SA stimulated the plant roots to secrete organic acids to acidify soil (Li et al., 2008). The different concentrations of SA increased the soil exchangeable Cd concentration; moreover, soil exchangeable Cd concentration was negatively correlated with soil pH. This observation is consistent with the previous study (Dou et al., 2020), which showed that beneficial for Cd uptake in *N. officinale*. SA decreased the Cd contents in roots and leaves of tomato and in various organs of *Populus deltoids × P. cathayana* under Cd stress (Wang, 2016; Wang et al., 2019). In rice, SA decreased the Cd accumulation in grains and ears at grain filling and maturity stages but increased the Cd accumulation in leaves at maturity stage. However, no significant impact of SA was observed on Cd accumulation in rice roots and stems (Liu, 2016). In this experiment, only 200 and 250 mg/L SA increased the *N. officinale* root Cd content, and 150, 200, and 250 mg/L SA increased the shoot Cd content. SA at 100, 150, and 200 mg/L concentrations increased the root Cd extraction and shoot Cd extraction. These results are consistent with the hyperaccumulators studies (Chen et al., 2011) but different from the reports on non-hyperaccumulators (Wang, 2016; Wang et al., 2019). Thus, our observations confirm that SA could promote the *N. officinale* Cd uptake for two reasons: (1) SA improved the *N. officinale* resistance to Cd, thus enhancing the Cd absorption capacity of *N. officinale*. Because the correlation analysis showed that both the root Cd and shoot Cd contents were positively correlated with the POD activity and CAT activity. (2) SA stimulated the plant roots to secrete organic acids to decrease the soil pH, thus increasing the soil available Cd concentration. Correlation analysis showed that both the root and shoot Cd contents were positively correlated with the soil exchangeable Cd concentration but were negatively correlated with the soil pH. Moreover, the grey relational analysis showed that the root Cd extraction, root biomass, and shoot biomass were the top three closely associated factors with the shoot Cd extraction. Therefore, the study indicated that the root Cd extraction, root biomass, and shoot biomass played an important role in determining the shoot Cd extraction capability (phytoremediation capability) of *N. officinale*.

## Conclusion

SA at 100, 150, and 200 mg/L concentrations promoted the *N. officinale* growth by increasing its biomass under Cd-contaminated conditions. Only 150 mg/L SA increased the chlorophyll *a* and *b* contents. Meanwhile, SA at different concentrations increased the POD activity, CAT activity, and Cd content to some extent, but decreased the SOD activity and soluble protein content. SA at 100, 150, and 200 mg/L increased the Cd extraction. In addition, the SA concentration showed a regression relationship with both the root and shoot Cd extractions, and the root Cd extraction, shoot biomass, root biomass had closely associated with the shoot Cd extraction. Thus, the study confirmed that SA could improve the *N. officinale* phytoremediation capability for Cd-contaminated paddy fields. However, future work should explore how SA promotes Cd accumulation in *N. officinale*.

## Data availability statement

The original contributions presented in the study are included in the article/supplementary material. Further inquiries can be directed to the corresponding author.

## Author contributions

YaZ, investigation, data curation, and writing- original draft preparation. RZ, YiZ, QA, HL, AL, and LW, investigation and data curation. LL, conceptualization and methodology. All authors contributed to the article and approved the submitted version.

## Funding

This work was supported by the Project of Shaanxi Provincial Science and Technology Department (2022ZY1-CGZY-07).

## Conflict of interest

The authors declare that the research was conducted in the absence of any commercial or financial relationships that could be construed as a potential conflict of interest.

## Publisher’s note

All claims expressed in this article are solely those of the authors and do not necessarily represent those of their affiliated organizations, or those of the publisher, the editors and the reviewers. Any product that may be evaluated in this article, or claim that may be made by its manufacturer, is not guaranteed or endorsed by the publisher.

## References

[B1] Anna M. Yelena L. Kristina G. Olga T. (2015). Effect of cadmium stress and inoculation with a heavy-metal-resistant bacterium on the growth and enzyme activity of *Sorghum bicolor* . Environ. Sci. pollut. R. 22 (20), 16098–16109. doi: 10.1007/s11356-015-4798-7 26066858

[B2] Azooz M. M. Youssef M. M. (2010). Evaluation of heat shock and salicylic acid treatments as inducers of drought stress tolerance in hassawi wheat. Am. J. Plant Physiol. 5 (2), 56–70. doi: 10.3923/ajpp.2010.56.70

[B3] Bai X. Dong Y. Kong J. Xu L. Liu S. (2015). Effects of application of salicylic acid alleviates cadmium toxicity in perennial ryegrass. Plant Growth Regul. 75 (3), 695–706. doi: 10.1007/s10725-014-9971-3

[B4] Bao S. (2000). Soil and agricultural chemistry analysis (Beijing, China: China Agriculture Press).

[B5] Chavoushi M. Najafi F. Salimi A. Angaji S. A. (2020). Effect of salicylic acid and sodium nitroprusside on growth parameters, photosynthetic pigments and secondary metabolites of safflower under drought stress. Sci. Hortic.-Amsterdam 259, 108823. doi: 10.1016/j.scienta.2019.108823

[B6] Chen Z. (2009). Effects of salicyic acid on seeds germination and seedlings growth of cauliflower under cadmium stress. Seed 28 (2), 39–43. doi: 10.3969/j.issn.1001-4705.2009.02.012

[B7] Chen Y. G. Chen K. N. Dai Q. Y. Kong Z. M. (2006). Metal enrichment of five soilless cultivated vegetables. J. Ecol. Rural Environ. 22 (1), 70–74. doi: 10.3969/j.issn.1673-4831.2006.01.016

[B8] Chen L. Long X. H. Jin L. Jin S. Z. Zhen X. T. Liu Z. T. (2011). Mitigation effects of exogenous salicylic acid on the seedling growth of two *Helianthus tuberosus* varieties under cd stress. Chin. J. Ecol. 30 (10), 2155–2164.

[B9] Dou W. An Y. Qin L. I. Lin D. Zeng Q. Xia Q. (2020). Advances in effects of soil pH on cadmium form. Soils 52 (3), 439–444. doi: 10.13758/j.cnki.tr.2020.03.002

[B10] Garg N. Singla T. Bhandri P. (2015). Metal uptake, oxidative metabolism, and mycorrhization in pigeonpea and pea under arsenic and cadmium stress. Turk. J. Agric. For. 39 (2), 234–250. doi: 10.3906/tar-1406-121

[B11] Guo B. Liang Y. C. Zhu Y. G. Zhao F. J. (2007). Role of salicylic acid in alleviating oxidative damage in rice roots (*Oryza sativa*) subjected to cadmium stress. Environ. pollut. 147 (3), 743–749. doi: 10.1016/j.envpol.2006.09.007 17084493

[B12] Guo S. C. Yang W. Q. (2015). Research progress of phytoremediation technology to remedicate heavy metal contaminated soil. J. Northwest For. Univ. 30 (6), 81–87. doi: 10.3969/j.issn.1001-7461.2015.06.14

[B13] Hao Z. B. Cang J. Xu Z. (2004). Plant physiology experiment (Harbin, China: Harbin Institute of Technology Press).

[B14] Kawano T. Bouteau F. (2013). Crosstalk between intracellular and extracellular salicylic acid signaling events leading to long-distance spread of signals. Plant Cell Rep. 32 (7), 1125–1138. doi: 10.1007/s00299-013-1451-0 23689257

[B15] Khan I. Seleiman M. F. Chattha M. U. Jalal R. S. Mahmood F. Hassan F. A. S.. (2021). Enhancing antioxidant defense system of mung bean with a salicylic acid exogenous application to mitigate cadmium toxicity. Not. Bot. Horti. Agrobo. 49 (2), 12303. doi: 10.15835/nbha49212303

[B16] Kováčik J. Grúz J. Bačkor M. Strnad M. (2009). Salicylic acid-induced changes to growth and phenolic metabolism in *Matricaria chamomilla* plants. Plant Cell Rep. 28 (1), 135–143. doi: 10.1007/s00299-008-0627-5 18972114

[B17] Krantev A. Yordanova R. Janda T. Szalai G. Popova L. (2008). Treatment with salicylic acid decreases the effect of cadmium on photosynthesis in maize plants. J. Plant Physiol. 165 (9), 920–931. doi: 10.1016/j.jplph.2006.11.014 17913285

[B18] Li C. J. Ma W. Zhang F. S. (2008). Rhizosphere talk and its impacts on plant growth. Plant Nutr. Fertilizer Sci. 14 (1), 178–183. doi: 10.3321/j.issn:1008-505X.2008.01.029

[B19] Lin L. J. Luo L. Miao M. A. Zhang X. Yang D. Y. (2015). Cadmium accumulation characteristics of emerged plant *Nasturtium officinale* r. BR. Resour. Environ. Yangtze Basin 24 (4), 684–689. doi: 10.11870/cjlyzyyhj201504021

[B20] Lin L. Wu C. Jiang W. Liao M. Tang Y. Wang J.. (2020). Grafting increases cadmium accumulation in the post-grafting generations of the potential cadmium-hyperaccumulator *Solanum photeinocarpum* . Chem. Ecol. 36 (7), 685–704. doi: 10.1080/02757540.2020.1760853

[B21] Liu Z. P. (2016). Heavy metal pollution in rice grain of the yangtze river and molecular and physiological mechanism of salicylic acid in regulating cadmium accumulation (Master thesis), (Hangzhou, China: China Jiliang University).

[B22] Liu L. Li X. Huang K. Zhu Y. Li A. Ao Q.. (2022). Salicylic acid promotes growth and affects cadmium accumulation of *Cyphomandra betacea* seedlings. Environ. Prog. Sustain. 41 (3), e13787. doi: 10.1002/ep.13787

[B23] Li Q. Wang G. Wang Y. Yang D. Guan C. Ji J. (2019). Foliar application of salicylic acid alleviate the cadmium toxicity by modulation the reactive oxygen species in potato. Ecotox. Environ. Safe. 172, 317–325. doi: 10.1016/j.ecoenv.2019.01.078 30721875

[B24] Ma Q. Fasih U. H. Muhammad F. Muhammad A. Noman S. Wu J.. (2022). Selenium treated foliage and biochar treated soil for improved lettuce (*Lactuca sativa* l.) growth in cd-polluted soil. J. Clean Prod. 335, 130267. doi: 10.1016/j.jclepro.2021.130267

[B25] Mahmoud A. E. D. Al-Qahtani K. M. Alflaij S. O. Al-Qahtani S. F. Alsamhan F. A. (2021). Green copper oxide nanoparticles for lead, nickel, and cadmium removal from contaminated water. Sci. Rep. 11 (1), 12547. doi: 10.1038/s41598-021-91093-7 34131155 PMC8206336

[B26] Majumdar S. Sachdev S. Kundu R. (2020). Salicylic acid mediated reduction in grain cadmium accumulation and amelioration of toxicity in *Oryza sativa* l. cv bandana. Ecotox. Environ. Safe. 205, 111167. doi: 10.1016/j.ecoenv.2020.111167 32827967

[B27] Pál M. Horváth E. Janda T. Páldi E. Szalai G. (2005). Cadmium stimulates the accumulation of salicylic acid and its putative precursors in maize (*Zea mays*) plants. Physiol. Plantarum. 125 (3), 356–364. doi: 10.1111/j.1399-3054.2005.00545.x

[B28] Pan J. Guan M. Xu P. Chen M. Cao Z. (2021). Salicylic acid reduces cadmium (Cd) accumulation in rice (*Oryza sativa* l.) by regulating root cell wall composition *via* nitric oxide signaling. Sci. Total Environ. 797, 149202. doi: 10.1016/j.scitotenv.2021.149202 34346363

[B29] Popova L. P. Maslenkova L. T. Yordanova R. Y. Lvanova A. P. Krantev A. P. Szalai G.. (2009). Exogenous treatment with salicylic acid attenuates cadmium toxicity in pea seedlings. Plant Physiol. Bioch. 47 (3), 224–231. doi: 10.1016/j.plaphy.2008.11.007 19091585

[B30] Riaz U. Aslam A. Uz Zaman Q. Sabiha J. Rehman G. Shazia L.. (2021). Cadmium contamination, bioavailability, uptake mechanism and remediation strategies in soil-plant-environment system: a critical review. Curr. Anal. Chem. 17 (1), 49–60. doi: 10.2174/1573411016999200817174311

[B31] Rostami S. Azhdarpoor A. (2019). The application of plant growth regulators to improve phytoremediation of contaminated soils: A review. Chemosphere 220, 818–827. doi: 10.1016/j.chemosphere.2018.12.203 30612051

[B32] Shaheen S. M. Rinklebe J. (2015). Impact of emerging and low cost alternative amendments on the (im)mobilization and phytoavailability of cd and Pb in a contaminated floodplain soil. Ecol. Eng. 74, 319–326. doi: 10.1016/j.ecoleng.2014.10.024

[B33] Shi G. Cai Q. Liu Q. Wu L. (2009). Salicylic acid-mediated alleviation of cadmium toxicity in hemp plants in relation to cadmium uptake, photosynthesis, and antioxidant enzymes. Acta Physiol. Plant 31 (5), 969–977. doi: 10.1007/s11738-009-0312-5

[B34] Shi Q. Zhu Z. (2008). Effects of exogenous salicylic acid on manganese toxicity, element contents and antioxidative system in cucumber. Environ. Exp. Bot. 63 (1), 317–326. doi: 10.1016/j.envexpbot.2007.11.003

[B35] Sofy M. R. Seleiman M. F. Alhammad B. A. Alharbi B. M. Mohamed H. I. (2020). Minimizing adverse effects of Pb on maize plants by combined treatment with jasmonic, salicylic acids and praline. Agronomy-Basel 10 (5), 699. doi: 10.3390/agronomy10050699

[B36] Song W. Chen S. Liu J. Chen L. Song N. Li N.. (2015). Variation of cd concentration in various rice cultivars and derivation of cadmium toxicity thresholds for paddy soil by species-sensitivity distribution. J. Integr. Agr. 14 (9), 1845–1854. doi: 10.1016/S2095-3119(14)60926-6

[B37] Song W. Y. Hong C. Y. Hong B. S. Ai Z. Z. Brestic M. (2014). The alleviative effects of salicylic acid on the activities of catalase and superoxide dismutase in malting barley ( *Hordeum uhulgare* l.) seedling leaves stressed by heavy metals. Clean-Soil Air Water. 42 (1), 88–97. doi: 10.1002/clen.201200310

[B38] Souza V. L. De Almeida A. F. Lima S. G. C. Cascardo J. C. M. Silva P. A. O. Gomes F. P. (2011). Morphophysiological responses and programmed cell death induced by cadmium in *Genipa americana* l. (Rubiaceae). BioMetals 24 (1), 59–71. doi: 10.1007/s10534-010-9374-5 20838856

[B39] Tamás L. Mistrík I. Alemayehu A. Zenova V. Bocava B. Huttova J. (2015). Salicylic acid alleviates cadmium-induced stress responses through the inhibition of cd-induced auxin-mediated reactive oxygen species production in barley root tips. J. Plant Physiol. 173, 1–8. doi: 10.1016/j.jplph.2014.08.018 25462072

[B40] Tang W. Xiao L. Peng X. Liu H. Zhu Y. Zheng Y. (2022). Effects of brassinolide on cadmium accumulation and growth of emerged accumulator plant *Nasturtium officinale* . Chem. Ecol. 38 (4), 301–311. doi: 10.1080/02757540.2022.2066086

[B41] Tissa S. Darren T. Eric B. Kingsley D. (2000). Acetyl salicylic acid (*Aspirin*) and salicylic acid induce multiple stress tolerance in bean and tomato plants. Plant Growth Regul. 30, 157–161. doi: 10.1023/A:1006386800974

[B42] Vatehová Z. Malovíková A. Kollárová K. Kucerova D. Liskova D. (2015). Impact of cadmium stress on two maize hybrids. Plant Physiol. Bioch. 108, 90–98. doi: 10.1016/j.plaphy.2016.06.035 27423219

[B43] Wang Z. M. (2016). The physiological regulation of exogenous salicylicacid on cadmium accumulation in populus × cathayana (Master thesis) (Yangling, China: Northwest A & F University).

[B44] Wang X. (2019). Application of grey relation analysis theory to choose high reliability of the network node. J. Phys.: Conf. Series. 1237 (3), 32056. doi: 10.1088/1742-6596/1237/3/032056/meta

[B45] Wang X. H. Guo J. K. Jia H. L. Li Y. P. Lv X. Ren Q. (2019). The effect of exogenous salicylic acid on alleviating cadmium toxicity in tomato plants. J. Agro-Environ. Sci. 38 (12), 2705–2714. doi: 10.11654/jaes.2019-0754

[B46] Wang W. Lin J. Zou H. Wu L. Huang W. Ju Y. (2009). Effects of salicylic acid on the ideotype and activities of antioxidant enzymes of *Narcissus ttazetta* l. var. chinensis roem. Chinese. Agric. Sci. Bull. 25 (14), 157–160.

[B47] Wang Z. Xia L. Song S. Farías M. E. Li Y. Tang C. (2021). Cadmium removal from diluted wastewater by using high-phosphorus-culturemodified microalgae. Chem. Phys. Lett. 771, 138561. doi: 10.1016/j.cplett.2021.138561

[B48] Wei S. H. Zhou Q. X. Wang X. Zhang K. S. Guo G. L. (2004). Solanum nigrum l — a newly discovered cd hyper-accumulator. Chin. Sci. Bull. 49 (24), 2568–2573. doi: 10.3321/j.issn:0023-074X.2004.24.013

[B49] Wu J. Chen Q. Tang C. Xia H. (2006). Effects of exogenous salicylic acid on lignification and related enzymes activities of loquat during cold storage. Trans. CSAE 22 (7), 175–179. doi: 10.3321/j.issn:1002-6819.2006.07.037

[B50] Xie L. J. Xu Z. R. (2003). The toxicity of heavy metal cadmium to animals and humans. Acta. Agri. Zhejiangensis 15 (6), 376–381. doi: 10.3969/j.issn.1004-1524.2003.06.012

[B51] Zawoznik M. S. Groppa M. D. Tomaro M. L. Benavides M. P. (2007). Endogenous salicylic acid potentiates cadmium-induced oxidative stress in *Arabidopsis thaliana* . Plant Sci. 173 (2), 190–197. doi: 10.1016/j.plantsci.2007.05.004

[B52] Zhang Y. T. (2018). Study on the molecular and physiologicalmechanisms of salicylic acid regulating cd accumulation in rice grain (Master thesis) (China: China Jiliang University).

[B53] Zhang S. R. Lin H. C. Deng L. J. Gong G. S. Jia Y. X. Xu X. X.. (2013). Cadmium tolerance and proline accumulation characteristics of *Siegesbeckia orientalis* l. Ecol. Eng. 51 (2), 133–139. doi: 10.1016/j.ecoleng.2012.12.080

[B54] Zhang X. Xia H. Li Z. Gao B. (2010). Potential of four forage grasses in remediation of cd and zn contaminated soils. Bioresource Technol. 101 (6), 2063–2066. doi: 10.1016/j.biortech.2009.11.065 20005700

[B55] Zhang X. F. Xia H. P. Li Z. A. Zhuang P. Gao B. (2011b). Identification of a new potential cd-hyperaccumulator *Solanum photeinocarpum* by soil seed bank-metal concentration gradient method. J. Hazard. Mater. 189 (1-2), 414–419. doi: 10.1016/j.jhazmat.2011.02.053 21397392

[B56] Zhang Y. Zhang L. Cheng H. Sun H. Cui X. (2020). Soil cadmium pollution and crop health risk in a mining area in south China. J. Agro-Environ. Sci. 39 (12), 2752–2761. doi: 10.11654/jaes.2020-0485

[B57] Zhang F. Zhang H. Xia Y. Wang G. Xu L. Shen Z. (2011a). Exogenous application of salicylic acid alleviates cadmium toxicity and reduces hydrogen peroxide accumulation in root apoplasts of *Phaseolus aureus* and *Vicia sativa* . Plant Cell Rep. 30 (8), 1475–1483. doi: 10.1007/s00299-011-1056-4 21409549

